# Advances in research on the role of neutrophils in organ transplant rejection

**DOI:** 10.3389/fimmu.2025.1677901

**Published:** 2025-10-27

**Authors:** Shaochen Yu, Mengjie Zhang, Ziyue Dou, Beibei Tian, Jian Lu

**Affiliations:** ^1^ Department of Emergency and Critical Care Medicine, Chuzhou Integrated Traditional Chinese and Western Medicine Hospital, Chuzhou, Anhui, China; ^2^ Department of Gastroenterology, The First Affiliated Hospital of Anhui Medical University, Hefei, Anhui, China

**Keywords:** neutrophil, neutrophil extracellular traps (NETs), organ transplantation, rejection, targeted therapy

## Abstract

Organ transplantation is an effective treatment for end-stage organ failure, but rejection remains a major obstacle to transplant success. Neutrophils play a key role in organ transplant rejection, participating not only in early immune responses but also exacerbating graft injury through mechanisms such as the release of neutrophil extracellular traps (NETs). Therefore, in-depth exploration of the immunological role of neutrophils in transplant rejection and their interactions with other immune cells is highly important. This article reviews the latest research progress on the mechanisms of action of neutrophils in transplant rejection and their impact on grafts while also assessing the clinical application prospects of immunosuppressive strategies targeting neutrophils and NETs. By integrating current basic and clinical research findings, this article aims to provide theoretical support and new research directions for the diagnosis and treatment of neutrophil-related rejection, with the goal of improving organ transplant success rates and patient quality of life.

## 1 Introduction

Organ transplantation is a critical treatment for end-stage organ failure, offering new hope to countless patients. However, posttransplant rejection remains one of the major challenges affecting graft survival and patient prognosis (1). The occurrence of rejection is closely related to adaptive immunity, but recent studies have shown that the innate immune system, particularly neutrophils, plays an increasingly important role in this process (2). Traditionally, organ transplant research has focused on adaptive immune mechanisms, but the critical role of neutrophils in graft immune injury is gradually being recognized.

As the first responders of the innate immune system, neutrophils are rapidly recruited to damaged sites following infection and tissue injury. Studies have shown that neutrophils not only directly participate in immune responses by releasing inflammatory mediators and forming extracellular traps but also regulate immune responses through interactions with other immune cells (3). The activation and function of neutrophils in organ rejection are particularly complex: they can promote rejection by enhancing inflammatory responses but may also promote graft tolerance under specific conditions (4, 5). This phenomenon suggests that a delicate balance between graft rejection and tolerance may exist for neutrophils. Some studies indicate that neutrophils promote acute transplant rejection while potentially playing a protective role in chronic rejection or tolerance states (6, 7).

This article systematically summarizes the latest research advances on neutrophils in organ transplant rejection, aiming to better understand the immunological mechanisms of their effects and provide new insights for clinical application. These findings suggest that therapeutic strategies targeting neutrophils may offer new directions and opportunities to mitigate rejection and improve graft survival, especially against the backdrop of the limited efficacy of current conventional immunosuppressive therapies (8, 9).

## 2 Immunological mechanisms of neutrophil action in organ transplant rejection

### 2.1 Mechanisms of neutrophil recruitment and activation

In organ transplantation, neutrophil recruitment and activation are crucial steps involving various proinflammatory factors. Studies have shown that proinflammatory cytokines such as interleukin (IL)-17 and complement C3 play significant roles in transplant rejection. IL-17, a cytokine produced by T cells, promotes the recruitment of neutrophils and enhances their migration to inflammatory sites (10). When activated, complement C3, a key component of the complement system, also promotes chemotaxis in neutrophils and enhances their response to pathogens (11). In transplant models, C3 activation leads to neutrophil aggregation, exacerbating graft inflammation (12). This process is mediated by specific chemokines (e.g., CXCL2) that guide neutrophil migration toward the graft (13, 14). This migration helps clear out potential pathogens but may also promote graft injury through the release of various cytokines and inflammatory mediators, ultimately affecting the survival and function of the graft.

Furthermore, Nuclear factor of activated T cells c3 (NFATc3) is an important transcription factor that plays a key role in neutrophil function. Research indicates that NFATc3 is involved not only in neutrophil development and differentiation but also in regulating their adhesion and migration processes (15). In inflammatory environments, NFATc3 activation promotes neutrophil adhesion to endothelial cells, thereby increasing their accumulation at inflammatory sites (2). This process is critical for the function of neutrophils, determining their ability to respond effectively to infection or tissue damage. Additionally, NFATc3 plays a significant role in regulating the tissue-destructive functions of neutrophils. During transplantation, NFATc3 activation is closely associated with neutrophil degranulation and inflammatory mediator release (2). The release of these inflammatory mediators can cause tissue damage and aggravate rejection, impacting long-term graft survival (16). Therefore, modulating NFATc3 activity may offer new therapeutic strategies to improve posttransplant inflammatory responses and reduce tissue damage.

### 2.2 Mechanisms of neutrophil-mediated tissue injury

Neutrophils are the body’s primary immune effector cells and play a vital role in infection and inflammatory responses. They participate in organ transplant rejection through multiple mechanisms, particularly by directly damaging graft cells through the release of reactive oxygen species (ROS) and proteases and the formation of NETs. Studies have shown that neutrophil activation and the release of pathogenic factors after transplantation are significant mechanisms contributing to graft injury (17).

The release of ROS is the primary way in which neutrophils respond to pathogens or tissue damage (18). Neutrophils rapidly accumulate at infection or injury sites and generate large amounts of ROS through NADPH oxidase activity. These ROS can directly attack cell membranes, proteins, and DNA, leading to cell damage and death (19–21). In grafts, excessive ROS production not only damages the graft cells but also triggers local and systemic inflammatory responses, exacerbating rejection (22, 23).

In addition to releasing ROS, neutrophils also exacerbate tissue damage by releasing various proteases. These proteases, including elastase and neutrophil gelatinase, can degrade extracellular matrix components, disrupt tissue structure, and cause irreversible functional damage to the graft (24, 25). Research has shown that protease release is closely associated with acute injury in lung transplantation, suggesting that proteases may be key therapeutic targets (26).

Furthermore, neutrophils damage grafts through the formation of NETs. NETs, which are composed of deoxyribonucleic acid (DNA) and antimicrobial proteins, trap and kill pathogens (27). However, excessive NET formation in the absence of infection leads to tissue damage. Regarding grafts in particular, NETs can directly adhere to graft cells, causing cell death and dysfunction (28, 29). These findings indicate that NETs play a dual role, participating not only in the defense against infection but also in immune rejection after organ transplant.

Thus, neutrophils directly damage graft cells through multiple mechanisms, including ROS production, protease release, and NET formation. These findings deepen our understanding of the role of neutrophils in transplant rejection and provide new insights and targets for future clinical treatments, particularly the development of immunomodulatory therapies targeting neutrophils (30).

## 3 Interactions between neutrophils and other immune cells

### 3.1 Neutrophils and adaptive immune cells

#### 3.1.1 Neutrophils and T cells

Recent research has shown that neutrophils actively regulate T-cell activation, differentiation, recruitment, and function through mechanisms such as antigen presentation, the secretion of key cytokines, and the release of NETs, playing a core regulatory role in driving and amplifying allograft rejection. In liver transplant rejection, activated neutrophils rich in mitochondrial DNA (mtDNA) significantly increase dendritic cell surface CD80/CD86-MHCII expression via the STING pathway, thereby driving CD8^+^ T-cell-mediated rejection (31). Simultaneously, the neutrophil-secreted chemokines CXCL9, CXCL10, and CXCL11 recruit effector T cells to the graft site by binding to the CXCR3 receptor on T cells, exacerbating tissue damage (31). In the tumor immune microenvironment, neutrophils release proinflammatory cytokines such as IL-12, IL-23, and IL-1β, inducing naïve CD4^+^ T cells to differentiate into proinflammatory subsets, promoting IFN-γ and IL-17 production, and thereby enhancing CD8^+^ T-cell cytotoxicity, forming a positive feedback loop that amplifies inflammation (32).

Notably, specific neutrophil subsets (e.g., CD177^+^ cells) further promote NET formation and inflammatory cytokine secretion by enhancing mitochondrial complex I activity, forming a vicious cycle in lung transplant reperfusion injury (33). Liao (34) et al. observed that Siglec-F^+^ neutrophils in the spleen exhibited immunosuppressive effects in the septic immune microenvironment by secreting IL-10, significantly inhibiting T-cell function and improving survival in infected mice. Wu (35) et al. utilized neutrophils costimulated with IL-23 and IL-18 (N(IL-23+IL-18)), which exhibit stronger T-cell stimulating capacity, significantly enhancing T-cell responses to allogeneic antigens. In a mouse skin transplant model, they found that adoptive transfer of graft- or host-derived N(IL-23+IL-18) significantly enhanced anti-donor antibody production, accompanied by an increase in splenic T follicular helper (Tfh) cells, thereby promoting tissue damage. Mechanistically, this effect partly stems from these neutrophils significantly overexpressing antigen-presentation-related genes in addition to MHC-II and costimulatory molecules.

**Figure 1:**
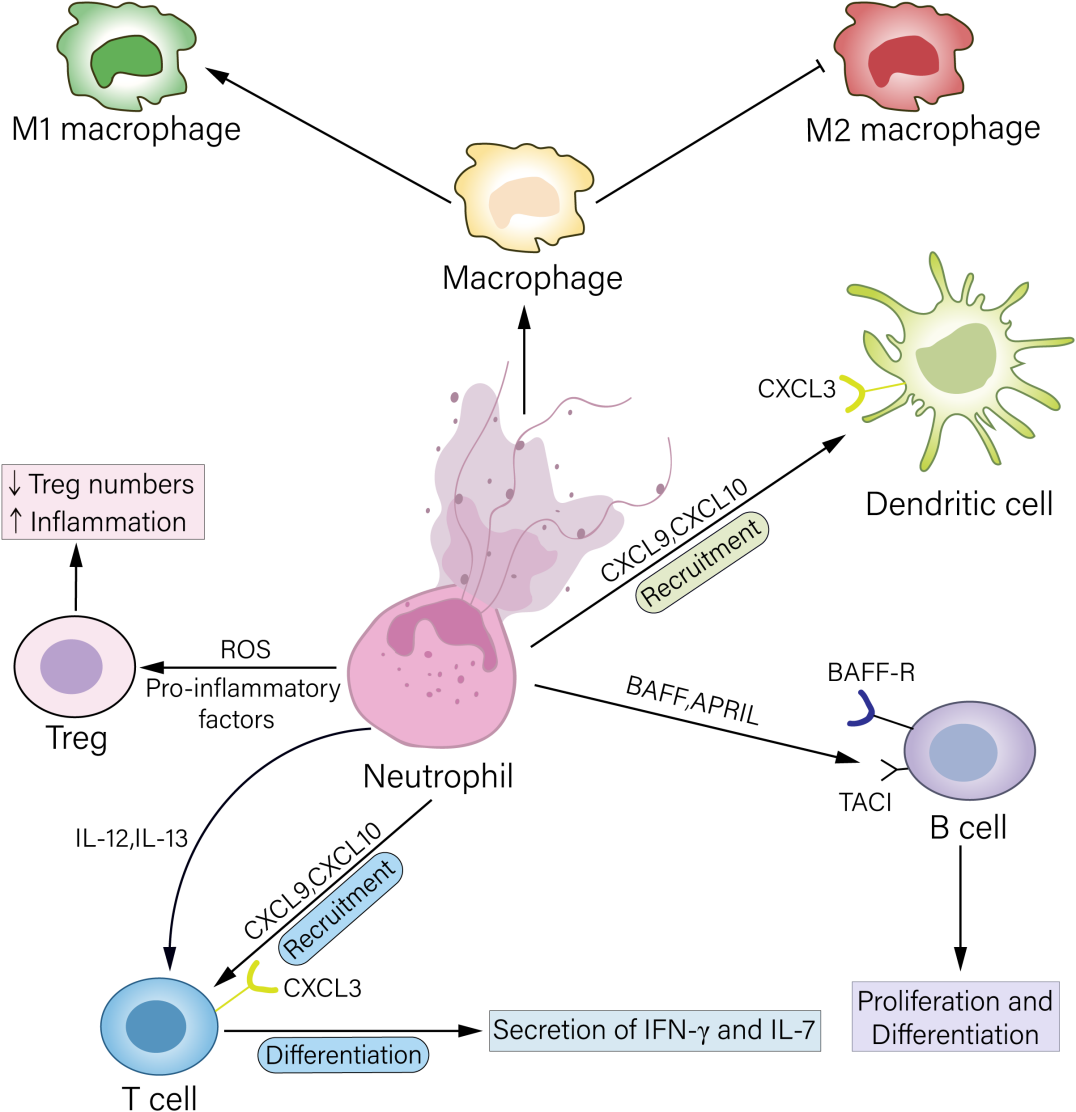
Mechanisms by which neutrophils regulate other immune cells. Neutrophils can act on Treg cells by releasing ROS or secreting proinflammatory factors, leading to a reduction in Treg cell numbers and thereby exacerbating inflammation. Neutrophils secrete the chemokines CXCL9 and CXCL10, which bind to the CXCR3 receptor on the surface of T cells and DCs, recruiting these cells to the inflamed site. Furthermore, neutrophils can secrete proinflammatory factors such as IL-12 and IL-23 to promote T-cell differentiation, thereby amplifying inflammation. Neutrophils highly express BAFF and APRIL, which bind, respectively, to the receptors BAFF-R and TACI on B-cells, regulating B-cell phenotypic switching and antibody secretion. NETs released by neutrophils can promote macrophage differentiation toward the M1 phenotype and inhibit macrophage differentiation toward the M2 phenotype, thereby promoting rejection.

With recent advances, the mechanisms by which neutrophils regulate regulatory T cells (Tregs) to participate in transplant rejection through multiple pathways are gradually being elucidated. In intestinal graft-versus-host disease (GVHD), neutrophils infiltrating the graft site activate local antigen-presenting cells by releasing ROS and proinflammatory cytokines (e.g., IL-6 and TNF-α), induce Th17 cell differentiation, and inhibit Treg expansion, worsening intestinal barrier function (36). Furthermore, neutrophil-derived interferon-γ (IFN-γ) and NETs can directly inhibit Treg activity, forming a positive feedback loop of “Treg deficiency → increased neutrophil infiltration → further impairment of Treg function,” leading to immune imbalance at the transplant site. Analysis of data from animal and clinical trials has revealed that a decreased Treg/neutrophil ratio is significantly correlated with reduced graft survival (**Figure 1**) (37, 38).

#### 3.1.2 Neutrophils and B cells

Neutrophils are not only effector cells involved in antibody-mediated rejection (AMR) but also core regulators that initiate and amplify humoral immunity (39, 40). They drive B-cell activation, plasma cell differentiation, and donor-specific antibody (DSA) production through multiple mechanisms, ultimately leading to graft microvascular damage. Research by the Harbin Medical University team reported high-density neutrophil infiltration in kidney tissue from a mouse model of AMR, with high expression of BAFF and APRIL (41). BAFF/APRIL binding to the B-cell surface receptors BAFF-R and TACI promotes B-cell proliferation, antibody class switching (e.g., IgG subtypes), and plasma cell differentiation (42). When the BAFF/APRIL signaling axis is blocked by the TACI–Fc fusion protein (Atacicept), DSA levels decrease, and plasma cell numbers are reduced (41, 42).

### 3.2 Neutrophils and innate immune cells

#### 3.2.1 Neutrophils and macrophages

Posttransplant graft loss is driven by communication between classical monocytes and tissue-resident nonclassical monocytes, prompting macrophages to release chemokines that recruit neutrophils to attack the graft. Damaged tissue triggers NET formation, further damaging the graft (43). NETs are special structures that form after neutrophil necrosis or apoptosis and are continuously involved in regulating immune and inflammatory responses (44). Liu (45) et al. reported that large quantities of NETs are released into the peripheral blood of liver transplant patients and that NET levels are closely related to the degree of rejection. In a rat liver transplant model, they found that NETs exacerbated rejection by stimulating Kupffer cell polarization toward the M1 phenotype. This M1 polarization can be mediated by NET components such as HMGB1, creating a pro-inflammatory feedback loop. Research in a haploidentical mouse bone marrow transplant model revealed that adoptive transfer of purified mature neutrophils from wild-type donor mice suppressed sterile and infectious sepsis in GVHD mice by regulating macrophage inflammatory responses via MMP9-mediated TGF-β1 activation (46). This illustrates an alternative, immunoregulatory pathway of neutrophil-macrophage crosstalk that can mitigate inflammation.

#### 3.2.2 Neutrophils and dendritic cells

In the transplant microenvironment, neutrophils actively coordinate dendritic cell (DC)-mediated adaptive immune responses through various contact-dependent and contact-independent mechanisms, thereby bridging innate recognition with graft rejection.

Neutrophils infiltrating the graft phagocytose donor-derived cellular debris and antigens. Subsequently, these neutrophils may undergo apoptosis and be engulfed by host dendritic cells—a process known as efferocytosis (16). Through this mechanism, donor antigens are transferred to dendritic cells, significantly enhancing their ability to present alloantigens and activate alloreactive T cells in secondary lymphoid organs and thereby initiating the rejection cascade.

In addition to serving as antigen carriers, neutrophils directly promote the maturation and functional activation of dendritic cells. As noted in section 3.1.1, the STING-dependent release of mtDNA by activated neutrophils in liver transplantation represents a key mechanism underlying this process. Upon uptake by DCs, mtDNA potently enhances their antigen presentation capacity, enabling the effective initiation of T cell responses (31).

Furthermore, in the inflammatory milieu, neutrophils secrete chemokines such as CXCL10 to establish a chemotactic gradient (47). This gradient recruits DCs and T cells to the site of inflammation by activating their CXCR3 receptor, thereby amplifying the local adaptive immune response.

## 4 Role of NETs in rejection

### 4.1 Mechanisms of NET formation

Neutrophils are crucial in the body’s immune defense, combating pathogen invasion through the release of NETs (48). NETs are web-like structures composed of extracellular DNA and antimicrobial proteins that trap and eliminate pathogens such as bacteria and fungi (49). In the context of organ transplantation, transplant-associated inflammatory stimuli are considered key factors that induce the production of NETs (50).

Studies have shown that neutrophils release NETs through various mechanisms in response to infection, inflammation, or tissue damage (51). For example, bacterial lipopolysaccharide (LPS) can induce NET formation by activating Toll-like receptor 4 (TLR4), a process often accompanied by ROS generation and inflammatory mediator release (52). Furthermore, research has shown that NET formation depends not only on ROS but also on other signaling pathways, such as the NF-κB and MAPK pathways. These pathways are activated in the early stages following neutrophil stimulation, promoting NET generation (53, 54).

**Figure 2:**
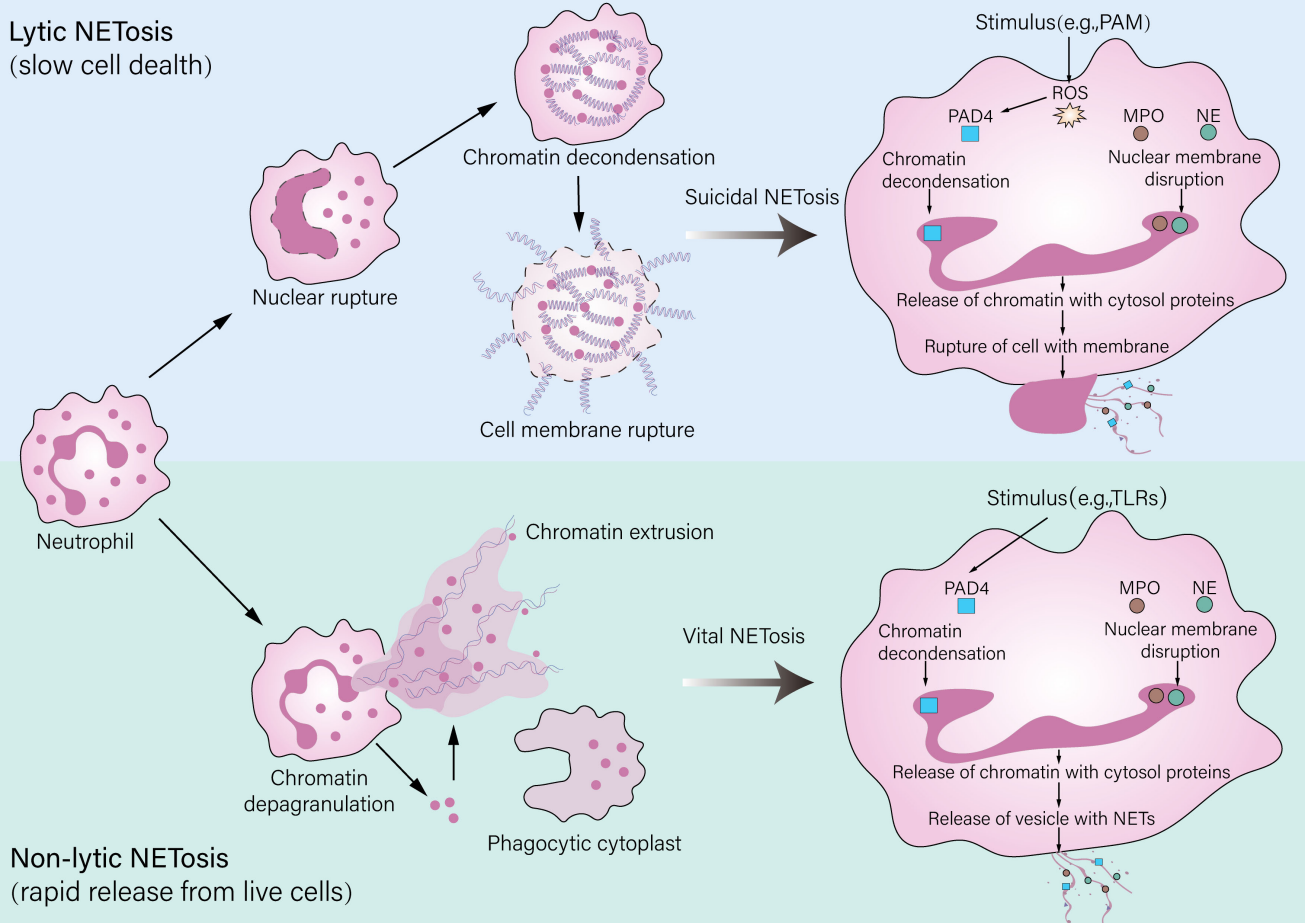
Pathways of neutrophil extracellular trap (NET) formation. Neutrophils release NETs through two distinct mechanisms: lytic and nonlytic NETosis. Lytic NETosis (also referred to as “suicidal NETosis”) is a slower process that leads to neutrophil death. It is characterized by rupture of the plasma membrane and the release of decondensed chromatin along with granular contents and can be triggered by stimuli such as PMA. Nonlytic NETosis (also known as “vital NETosis”), by contrast, is a rapid mechanism that enables NET release from living neutrophils and may involve the formation of a phagocytic cytoplast. This pathway can be initiated by stimuli including LPS, pathogens via TLR activation, or interactions with platelets.

NET formation occurs primarily through two distinct pathways: “suicidal” NETosis and “vital” NETosis (55). In suicidal NETosis, potent stimuli such as phorbol myristate acetate (PMA) trigger a robust ROS burst from NADPH oxidase, leading to the disintegration of the nuclear and granular membranes; this is followed by peptidylarginine deiminase 4 (PAD4)-mediated chromatin decondensation and the mixing of nuclear DNA with granule proteins like neutrophil elastase (NE) and myeloperoxidase (MPO), culminating in the lytic death of the neutrophil and thus the extracellular release of NETs (56). In contrast to suicidal NETosis, vital NETosis allows neutrophils to release NETs in the absence of immediate lytic death through a pathway initiated by specific bacteria like *Staphylococcus aureus* or activated platelets via receptors such as TLR2, which may bypass extensive ROS production and involves the vesicular release of nuclear or mitochondrial DNA (57, 58). Crucially, neutrophils undergoing vital NETosis can retain chemotactic and phagocytic functions, enabling them to continue participating in immune responses (59). The specific pathway activated is context-dependent and influences the magnitude and persistence of the NET response during the inflammatory process (**Figure 2**).

During transplantation, factors such as graft ischemia/reperfusion injury and immune rejection can trigger strong inflammatory responses, stimulating neutrophil activation and NET release (28). Clinical data have shown that the degree of rejection in liver transplant patients is positively correlated with NET levels, suggesting that more severe graft injury may lead to more NET release, forming a positive feedback loop that exacerbates local inflammation (28). Moreover, studies have shown that NET formation is closely related to delayed neutrophil apoptosis; this delay not only increases the quantity of NETs but also may cause persistent tissue damage and inflammation (60).

The involvement of renal tubular epithelial cells (TECs) in NET-mediated injury represents a novel and critical mechanism of solid organ ischemia-reperfusion injury, particularly in the kidney (61). Activated neutrophils and NETs can directly induce TEC death (62). By acting as damage-associated molecular patterns (DAMPs), NET components such as histones and cell-free DNA can activate pattern recognition receptors (e.g., TLR4) on TECs, thereby prompting the secretion of potent neutrophil chemoattractants like CXCL1 and CXCL2, which recruit more neutrophils and perpetuate the cycle of NET formation and tubular injury (63).

### 4.2 Impact of NETs on grafts

NETs play an important role in organ transplant rejection (45). However, excessive NETs not only combat infection but also have the ability to cause vascular endothelial damage and activate the complement system, thereby inducing antibody-mediated rejection (AMR) (64). In kidney transplantation, NETs activate the complement system, promote complement component deposition, and initiate and exacerbate local inflammation, forming a vicious cycle that leads to further graft damage and aggravated rejection (65). Li (66) et al. reported that NETs in the kidney transplant microenvironment promote increased ferrous ion content via the ERK1/2 pathway, activating iron metabolism-related protein expression and thereby causing graft dysfunction. In acute kidney rejection, intrarenal cytotoxic NETs can promote platelet activation; the granular contents released by activated platelets promote the secretion of proinflammatory cytokines and cytotoxic molecules, enhancing the inflammatory effects in the transplant microenvironment (67, 68).

Similarly, during lung transplantation, NETs play a role in promoting vascular damage. Research has shown that NETs can bind to vascular endothelial cells via von Willebrand factor (vWF) and P-selectin, providing a scaffold for platelet aggregation, which leads to fibrin deposition and thrombotic microangiopathy (69). During liver ischemia/reperfusion injury, DAMPs in the inflammatory environment induce NET formation by activating TLR signaling pathways, thereby exacerbating graft injury (70).

Furthermore, the pathological accumulation of NETs is not only a result of NET overproduction but also a consequence of impaired NET clearance. Under physiological conditions, NETs are efficiently dismantled to prevent persistent inflammation and tissue damage. The primary clearance mechanism involves the degradation of extracellular DNA by serum deoxyribonuclease I (DNase I), while macrophages, particularly those with an anti-inflammatory phenotype, contribute to the phagocytic removal of NETs (71). However, in the context of transplantation, this balance is disrupted. The inflammatory microenvironment can inhibit DNase I activity (72), and the polarization of macrophages towards a proinflammatory M1 phenotype can impair their ability to clear NETs (73). This failure in clearance leads to the persistence of NETs in the graft, which continue to act as a reservoir of DAMPs, thereby perpetuating a vicious cycle of neutrophil recruitment, and sustained inflammatory injury (74).

Therefore, NETs negatively impact graft outcomes in organ transplantation through various pathways, including promoting vascular endothelial damage, activating the complement system, and activating platelets, as well as through their persistence due to impaired clearance. Therapeutic strategies targeting NETs may become a potential approach to improve transplant outcomes. Inhibiting the formation of NETs or promoting their degradation and clearance could help reduce the incidence of rejection and improve graft survival.

### 4.3 Impact of NETs on acute and chronic rejection

The roles of NETs in acute and chronic rejection differ significantly. In acute rejection, NET formation is typically rapid and substantial; studies have shown that NET accumulation is closely associated with the occurrence of acute rejection. NETs rapidly trigger tissue damage by trapping and killing pathogens while promoting inflammatory responses (63). For example, in kidney transplantation, the presence of NETs is considered an important driver of acute rejection; their formation is closely related to endothelial damage and cytokine release, leading to increased inflammation and accelerated rejection (4).

In contrast, the role of NETs in chronic rejection is more complex. Chronic rejection is typically manifested as a long-term immune response and persistent tissue damage; NETs may act by promoting chronic inflammation and fibrosis processes (44, 75). To date, there has been relatively little research on NETs in chronic rejection. Luo (76) et al. reported that, after liver transplantation, NETs may promote the development of biliary atresia by modulating immune cell activity in the local microenvironment, as reflected by increased expression of profibrotic tissue factors and IL-17.

In summary, the differing roles of NETs in acute and chronic rejection reflect their dual role in graft immune surveillance and tissue damage. Interventions targeting NETs may need to be personalized according to the type and stage of rejection to achieve optimal transplant outcomes.

### 4.4 NETs as potential therapeutic targets

During organ transplantation, excessive NET formation leads to graft damage and rejection; therefore, inhibiting NET formation may be a new approach to reduce early posttransplant rejection. For example, in recipients of lung transplants, Lindstedt (77) et al. used cytokine adsorption to reduce graft damage and improve function. This treatment primarily reduces NET generation, thereby lowering inflammation, mitigating organ damage, and ultimately improving graft survival. In kidney transplantation, Pei (4) et al. identified four NET-related biomarkers during acute rejection; among these biomarkers, GPX3 expression was negatively correlated with the degree of acute rejection. Further animal experiments revealed that inhibiting GPX3 reduced the activation of NETs and the release of inflammatory factors, ultimately improving graft function.

In terms of clinical application, although more clinical trials are needed to verify the efficacy and safety of NET inhibitors, preliminary studies have already demonstrated their broad potential for use in skin grafting (78). Regulating NET formation could provide new therapeutic options for posttransplant patients, reducing the incidence of rejection and improving long-term graft survival. Therefore, therapeutic strategies targeting NETs may become an important direction for future organ transplant research and clinical practice.

## 5 Neutrophil functional heterogeneity and its precise regulation

As neutrophils are the most abundant leukocytes in the immune system, their functional heterogeneity is gradually attracting increased attention from researchers. Recent studies indicate that neutrophils are not a homogeneous population but rather consist of multiple subsets, which exhibit unique functions and mechanisms of action in different physiological and pathological states (79). In organ transplant rejection, this heterogeneity is particularly significant, with different neutrophil subsets playing different roles in rejection (35, 80).

First, research has shown that neutrophil function can change on the basis of their activation state in different microenvironments. In the context of acute inflammation or transplantation, the function of neutrophils can be divergent, with these cells exerting either protective or detrimental effects. On one hand, in the context of acute inflammation, mature neutrophils are rapidly recruited to damaged tissues to perform beneficial functions such as phagocytosis, ROS production, and the release of cytotoxic substances to combat infection and clear out dead cells (81). Some neutrophil subsets can also promote healing; for example, one study revealed that CD49d^+^ neutrophil accumulation promoted vascular remodeling at skin transplant sites in mice and shifted macrophages and dendritic cells toward a pro-regenerative phenotype, thereby improving posttransplant skin healing (82). On the other hand, in the context of rejection or injury, other subsets may become overactivated, leading to tissue damage and worsened inflammation, i.e., “hyperinflammation” (83). For instance, OLFM4^+^ neutrophils are primarily involved in the pathological processes of intestinal barrier disruption and death after reperfusion in mouse intestinal ischemia/reperfusion injury (84). Similarly, in the context of alcohol-associated hepatitis, high-density neutrophils (HDNs) release more NETs, resulting in liver damage, whereas low-density neutrophils (LDNs) exhibit a functionally defective state (18).

Second, gene expression profiles differ significantly between neutrophil subsets, indicating their distinct functions in responding to different types of injury and regulating immune responses. Some subsets exhibit stronger phagocytic capacity and ROS production in response to specific bacterial infections, whereas others may primarily participate in modulating inflammatory responses and promoting tissue repair (85). This functional diversity makes the role of neutrophils in transplant rejection more complex.

Furthermore, complex interactions of signals within the microenvironment can regulate neutrophil subset differentiation and function. The key factors shaping neutrophil functional transitions include inflammatory mediators, cytokines, and tissue-specific signals, which are the main drivers of neutrophil polarization and functional reprogramming (86–88). Similar to the M1/M2 polarization states of macrophages (89), neutrophils can polarize into the N1 and N2 subtypes, which exert proinflammatory and anti-inflammatory effects, respectively (90). N1-type neutrophils polarized by inflammatory factors such as LPS/IFN-γ show increased expression of surface markers such as CD11b, CD66b, and CD64 (86, 91), whereas N2-type neutrophils polarized by anti-inflammatory factors such as TGF-β/IL-4 exhibit increased levels of CD16, CD163, and CD206, often presenting an immunoregulatory phenotype (92, 93). These findings suggest that specific cytokines can promote the transition of neutrophils toward specific functional states, influencing their role in transplant rejection. This dynamic regulation provides potential clinical targets; precise regulation of neutrophil function holds promise for improving posttransplant rejection (94). Currently, in oncology, researchers are exploring the use of specific drugs or biologics to selectively modulate neutrophil function for antitumor effects (95). In the future, this precise therapeutic approach could be adapted to other fields, potentially improving transplant success rates and offering new treatment directions for other neutrophil-related diseases.

## 6 Safety and efficacy evaluation of neutrophil-targeted therapies

In recent years, therapies targeted against neutrophils and their role in organ transplant rejection have gained increasing attention. Evaluating the efficacy and safety of these novel treatment approaches, especially concerning immune balance, is a research focus. Neutrophils not only play a crucial role in rejection but also bear a close relation to posttransplant infections and other complications. Therefore, while targeting NETs may improve organ transplant outcomes, it may also have potential side effects.

Neutrophil-targeted therapeutic strategies include the use of monoclonal antibodies and targeted small molecules, among others. These therapies can effectively inhibit neutrophil activity (96, 97), potentially mitigating rejection. Leitch (98) et al. reported that the inhibitor ABT-737, which targets the apoptosis gene Bcl-2 family, promoted neutrophil apoptosis and reduced lung ischemia/reperfusion-related tissue damage. In animal models of ischemia/reperfusion injury, the CXCR2 antagonist navarixin has shown promising results in reducing neutrophil infiltration and associated tissue damage (99, 100). However, these treatments may also cause overall immune system suppression, increasing the risk of infection. For example, NET-targeted therapies might impair immune surveillance, preventing timely clearance of potential pathogens and leading to infectious complications (101). On the other hand, concerning immune balance, NET-targeted therapies need to find an equilibrium between suppressing rejection and maintaining the body’s immune function. Excessive neutrophil suppression may reduce anti-infection capacity, whereas insufficient suppression may fail to effectively control rejection. Therefore, it is crucial to develop individualized treatment plans, which require adjustment of therapeutic strategies and drug doses on the basis of the patient’s specific condition to achieve optimal efficacy and safety.

Furthermore, the side effects of targeted therapies must be considered. Research indicates that targeted therapies may be associated with various adverse reactions, including liver and kidney function impairment, allergic reactions, and other immune-related side effects. These side effects not only affect patient quality of life but also may limit treatment continuity and efficacy (102, 103). Therefore, when neutrophil-targeted therapy is implemented, a comprehensive assessment of the patient’s overall condition, including underlying diseases, immune status, and prior treatment history, is essential to ensure treatment safety and efficacy.

In conclusion, novel targeted therapies show promise for the treatment of neutrophil-related organ transplant rejection, but their safety and efficacy evaluation still require further research and clinical trial support. Future research should focus on optimizing targeted treatment regimens and finding suitable biomarkers to monitor treatment response, with the goal of achieving better immune balance and patient outcomes.

## 7 Conclusion

The role of neutrophils in organ transplant rejection, particularly their critical role in immunomodulation and tissue damage, is receiving increasing attention. Research indicates that neutrophils exacerbate graft damage through NET release, providing a new perspective for understanding transplant rejection mechanisms (50). NET formation not only promotes local inflammation but also may affect immune cell activation and recognition of the graft, thereby aggravating rejection (104).

However, differing views persist regarding the dual role of neutrophils in transplant rejection, in which they serve as protective immune cells while also causing tissue damage. This phenomenon reflects the heterogeneity and complexity of neutrophil function. Some studies suggest that neutrophils may play a protective role during early rejection, helping clear out pathogens and promote wound healing, whereas in later stages, their overactivation may lead to immune damage (105–107). Balancing these different findings is currently a pressing issue in the field.

Future research should pay further attention to the functional heterogeneity of neutrophils, aiming to achieve effective prevention and treatment of transplant rejection through precise immune regulation. Conducting more detailed research on different neutrophil subsets and their functional states will provide crucial evidence for clinical translation. By analyzing neutrophil responses under various conditions and combining high-throughput technologies and single-cell sequencing, it may be possible to reveal their specific mechanisms of action in transplant rejection (79).

Advancing research in this field will not only deepen the understanding of the role of neutrophils in transplant rejection but also provide theoretical support for clinical applications. In the future, precise regulation of neutrophil function may lead to new therapeutic strategies, improving transplant patient prognosis and success rates. In-depth research into the role of neutrophils in organ transplantation will not only advance transplant immunology but also lay the foundation for safer transplant protocols.
